# Evidence on the therapeutic role of thiolutin in imiquimod‐induced psoriasis‐like skin inflammation in mice

**DOI:** 10.1002/iid3.877

**Published:** 2023-07-12

**Authors:** Aixue Wang, Xixing Ma, Feng Wei, Yanling Li, Qiang Liu, Huanhuan Zhang

**Affiliations:** ^1^ Department of Dermatology The Second Affiliated Hospital of Hebei Medical University Shijiazhuang Hebei China

**Keywords:** inflammation, NLRP3, psoriasis, thiolutin

## Abstract

**Introduction:**

A recent study confirmed that thiolutin (THL), as a potent inflammasome inhibitor, plays a promising therapeutic role in multiple inflammatory disease models. However, the effect of THL on psoriasis has not been reported so far.

**Methods:**

A psoriasiform dermatitis model was prepared by applying 5% imiquimod (IMQ) cream on mice. A total of 36 mice were randomly divided into six groups: control, model, model + THL‐L/M/H (THL, 1/2.5/5 mg/kg/day), model + methotrexate (1 mg/kg/day). Psoriasis area and severity index (PASI) scores were observed and calculated. The histological changes in skin, liver, and kidney tissues were observed by hematoxylin and eosin staining. Alanine aminotransferase, aspartate aminotransferase, blood urea nitrogen, and blood creatinine were measured by automatic biochemistry analyzer. The size of the spleens was determined, and the proportion of Foxp3 + CD4+ regulatory T (Treg) cells in the spleens was tested by flow cytometry. The proinflammatory factors and nucleotide oligomerization domain nucleotide oligomerization domain (NOD)‐like receptor protein 3 (NLRP3) inflammasome protein levels were examined by reverse transcription‐quantitative polymerase chain reaction, enzyme‐linked immunosorbent assay, Western blotting, and immunohistochemistry, respectively.

**Results:**

THL administration preeminently reduced the thickness, scaling, and erythema of the skin lesions, alleviated IMQ‐induced psoriasiform lesions in mice, reduced the PASI score, and ameliorated histopathological changes in mouse skin. The spleen index was decreased by almost half and the proportion of Foxp3 + CD4+ Treg cells was increased after intervention by THL. THL intervention did not affect liver and kidney function, but decreased the expression levels of proinflammatory factors and NLRP3 inflammasome in the skin of psoriatic mice.

**Conclusions:**

THL may alleviate IMQ‐induced psoriasis‐like manifestations in mice by inhibiting NLRP3 inflammasome.

## Graphical abstract

Studies speculated that NLRP3 inflammasome has a role in the pathogenesis of IMQ‐induced psoriasis‐like lesions in mice, and THL may alleviate psoriasis‐like manifestations in mice by inhibiting NLRP3 inflammasome. The study herein provides a new drug candidate for the treatment of psoriasiform skin inflammation, and sheds new light on the subsequent research of psoriasis.

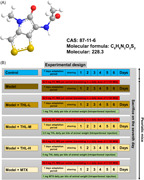

## 1 INTRODUCTION

Psoriasis is a common chronic inflammatory immune‐mediated skin disorder typically induced by a variety of environmental and genetic factors, which can occur anywhere on the skin, typically involving the forearm, periumbilical, perianal, retro‐auricular areas, and the scalp.^1^ Currently, the worldwide prevalence of psoriasis accumulates to 2%–3%.^2^ Most types of psoriasis have a periodic evolution with regression after a few weeks or months of onset, such that the stepwise onset causes substantial distress to the quality of life of the affected population, and this visible intractable skin disease also imposes a significant psychological burden on patients.^3^ Psoriasis is classified into five types, including plaque psoriasis (also known as psoriasis vulgaris), punctate or eruptive psoriasis, retro‐lenticular psoriasis (also known as trigeminal or ectodermal psoriasis), pustular psoriasis, and erythrodermic psoriasis.^4^ Psoriasis vulgaris is the most common of these, whereas all psoriatic phenotypes share symptoms including itching, burning, and pain.^5^ The pathologic features of psoriasis are mainly red plaques with white multilayered scales, acanthotic thickening of the epidermis, hyperkeratosis (thickening of the cornified layer) and parakeratosis (with nuclei in the keratinized layer), marked elongation of the epidermal rete ridges, dilated and distorted blood vessels extending to the apical end of the dermal papilla, and increased numbers and accumulation of inflammatory cells, macrophages, and neutrophils containing T lymphocytes in the dermal and epidermal layers.^6^ However, the exact pathogenesis of psoriasis remains undefined, but it is generally believed that the inflammatory response and abnormal activation of immune cell function play crucial roles in the pathogenesis and development of psoriasis.^7^ A plethora of inflammatory factors results in a dramatic increase in cytokines released from immune cells, and the epidermal symptoms are significantly enhanced, which eventually leads to the aggravation of psoriasis.^8^

The commonly used topical therapies for patients with psoriasis mainly include chemical drugs, such as glucocorticoids, vitamin D3 derivatives, tretinoin, dithranol, and so forth, the most commonly used of which are glucocorticoids.^9^, ^10^ In addition to the chemical class of drugs, there are many biological agents, including calcineurin inhibitors, methotrexate (MTX), cyclosporine, tumor necrosis factor (TNF) inhibitors, and interleukin (IL) inhibitors, among others.^11^ The requirements of existing therapies for the safe and effective treatment of psoriasis are unmet. Traditional Western medicines are not suitable for long‐term use because of limited efficacy and potential toxic side effects, whereas new biologics have not been widely used because of the high price and uncertainty regarding adverse effects, long‐term efficacy, and safety.^12^, ^13^ As the existing treatments often have side effects, they can only achieve suppression of the condition, but cannot completely cure it. Therefore, there is an urgent clinical need for drugs with therapeutic effects on psoriasis.

The nucleotide oligomerization domain (NOD)‐like receptor protein 3 (NLRP3) inflammasome, which has attracted attention in recent years, is an intracellular pattern recognition receptor (PRR) of the innate immune system.^14^ It is known that multiple danger signals can promote caspase‐1‐dependent production of inflammatory factors such as IL‐1β and IL‐18 via the NLRP3 inflammasome, which further cause immune cell activation such as T cells and natural killer cells, and release of inflammatory factors such as IFN‐γ and TNF‐α, which play important roles in the development of psoriasis.^15^ In the past decade, an increasing number of studies have confirmed that inflammasome activation is involved in the development and progression of psoriasis. Deng et al.^16^ discovered that NLRP3 inflammasomes were significantly activated in the stratum corneum of psoriatic dermatitis induced by imiquimod (IMQ), and inhibiting the expression of NLRP3 could improve psoriatic dermatitis in mice. Luo et al.^17^ suggested that NLRP3 expression was preeminently upregulated in an in vivo model of psoriasis compared with normal tissues, and NLRP3 inflammasome inhibitor treatment ameliorated psoriasis‐associated inflammation in mice. These data suggest that inflammasomes could be used as a novel therapeutic target for the treatment of psoriasis.

Thiolutin (THL), a sulfur‐containing antibiotic, is a potent inhibitor of bacterial and yeast RNA polymerases.^18^ THL was demonstrated to be an intracellular zinc chelator capable of inhibiting the formation of the BRCC3‐containing isopeptidase complex that is involved in NLRP3 inflammasome activation.^18^ BRCC3 can directly bind to NLRP3 and favors NLRP3 inflammasome activation through its deubiquitination.^19^ Recently, THL was confirmed to be a potent inflammasome inhibitor with promising therapeutic effects in multiple inflammatory disease models. Ren et al.^20^ revealed that THL treatment attenuated NLRP3‐related diseases such as sepsis, monosodium urate‐induced peritonitis, and mice with nonalcoholic fatty liver disease. However, its effect on psoriasis has not been reported so far. Considering that multiple reports highlight the potential of NLRP3 inflammasome as therapeutic target for psoriasis, we wonder whether THL exerts a promising therapeutic effect on psoriasis by regulating NLRP3 inflammasome.

In this study, a mouse psoriasis‐like model was established by topical IMQ administration, and we observed the effects of different doses of THL on the psoriatic area, liver and kidney function, spleen index, inflammatory factor levels, and NLRP3 inflammasome expression in an IMQ‐induced mouse psoriasis‐like model. This study sought to understand whether there was a good effect of THL on the treatment of psoriasis, hoping to provide a better drug option for the treatment of psoriasis.

## 2 METHODS

### 2.1 Experimental animal

Thirty‐six male BALB/c mice (8–10 weeks old) were purchased from the animal experimental center of Guangzhou Ruige Biotechnology Co., Ltd. Rearing conditions: daily light was maintained, temperature 25 ± 2°C, and relative humidity 55 ± 5%. Sufficiently cited tap water to ensure a nutritional balance of the mouse diet. All animal experimental protocols were reviewed and approved by the institutional animal care committee of the Second Affiliated Hospital of Hebei Medical University. Mice were housed for 1 week after acclimatization for experiments.

### 2.2 Establishment of a psoriasis model

The mice were randomly divided into six groups (*n* = 6) as follows: control, model, model + THL‐L, model + THL‐M, model + THL‐H, and model + MTX groups. For the control group, after the mice were acclimated to feeding for 7 days, the hair of around 8 cm^2^ size on the dorsal skin of the mice was removed to leave the skin exposed, and subsequently the mice were normally fed without any treatment. For the model group, after 7 days of adaptive feeding, mice were removed about 8 cm^2^ of hair, and then 62.5 mg of 5% IMQ was smeared on the exposed skin on the back of mice every day for 6 consecutive days to establish psoriasis model. For the model + THL‐L groups, based on the IMQ model, 1 mg/kg of THL (Tocris Bioscience) was intraperitoneally injected into mice daily. For the model + THL‐M groups, based on the IMQ model, 2.5 mg/kg of THL was intraperitoneally injected into mice daily. For the model + THL‐H groups, based on the IMQ model, 5 mg/kg of THL was intraperitoneally injected into mice daily. For the model + MTX group, based on the IMQ model, 1 mg/kg of MTX (Sigma‐Aldrich) was intraperitoneally injected into mice daily as a positive control for THL. At Day 7, all mice were killed by cervical dislocation after anesthesia with 1% sodium pentobarbital intraperitoneal injection (30 mg/kg). The dorsal bare skin of mice was excised and cleaned into physiological saline, and subsequently immediately put into 4% paraformaldehyde solution for fixation for making paraffin sections. The spleens, livers, and kidneys of the mice were removed and placed into normal saline for washing and then stored at −80°C for further use.

### 2.3 Psoriasis area and severity index (PASI) scores in mice and phenotypic observation

The mice were examined macroscopically for the extent of scaling, erythema, and hypertrophy of the skin on their back and processed for PASI scoring. The severity of psoriasis was evaluated from three perspectives: erythema, scale, and thickness. Scoring criteria: 0—none; 1—slight; 2—moderate; 3—marked; 4—very marked.

### 2.4 Mouse spleen index assay

On the day of mice sacrifice, mouse spleens were stripped and weighed separately. Spleen index = mouse spleen weight (mg)/mouse body weight (g).

### 2.5 Hematoxylin and eosin (HE) staining

Skin, liver, and kidney tissues from mice in each group were deparaffinized (xylene treatment for 5 min), hydrated (treated sequentially in 100% ethanol twice, 95% ethanol, 85% ethanol, and 75% ethanol for 30 s each), immersed in distilled water, and stained for nuclei (hematoxylin staining for 3 min), washed in tap water and stained for nuclei (0.5% HCl in ethanol for 10 s), rewashed in tap water and stained for cytoplasm (1% eosin immersion staining for 3 min), washed in tap water, dehydrated, and transparent. The sections were finally placed under a microscope for observation and images were acquired.

### 2.6 Immunohistochemistry

Tissue sections from mouse skin were deparaffinized, hydrated, antigen retrieval, serum blocking, incubation of primary antibody (NLRP3; Proteintech) and corresponding secondary antibodies, washing, diaminobenzidine color development, hematoxylin counterstaining, and mounting of the sections were performed sequentially. The expression and localization of NLRP3 in skin tissues were detected in three randomly selected high‐power fields per section under a microscope.

### 2.7 The proportion of regulatory T (Treg) cells was determined by flow cytometry

Spleens from mice in each group were removed to remove surrounding excess tissue, which was triturated, digested, filtered through a 70 mm mesh, and centrifuged to obtain a single‐cell suspension. Phorbol 12‐myristate 13‐acetate, brefeldin A, and lonomycin (Sigma‐Aldrich) were added to the cells, and incubation was continued at 37°C in 5% CO_2_. The fluorescein‐labeled monoclonal antibody CD4‐FITC (Cell Signaling Technology) was then added to incubate in the dark at room temperature. After treatment with the fixative for ruptured nuclear membrane, the intracellular antibody FOXP3‐APC was added. Finally, data were acquired with detection in flow cytometry.

### 2.8 Biochemical parameter assay

Biochemical parameters, including alanine aminotransferase (ALT), aspartate aminotransferase (AST), blood urea nitrogen (BUN), and blood creatinine (CREA), were measured in mouse serum samples using a fully automated biochemical analyzer (AU400; Olympus) according to the manufacturer's instructions (Oulebo Medical devices).

### 2.9 Enzyme‐linked immunosorbent assay (ELISA)

The levels of TNF‐α (Thermofisher), IL‐1β (Thermofisher), IL‐6 (Thermofisher), and IL‐17 (Thermofisher) were measured by enzyme‐linked immunosorbent assay, and the experimental procedures were strictly performed according to the kit instructions.

### 2.10 Reverse transcription‐quantitative polymerase chain reaction (RT‐qPCR)

The skin tissues of mice in each group were collected, the RNA concentration of each sample was determined, and the RNA was reverse transcribed into complementary DNA (cDNA) following the reverse transcription kit protocol. This cDNA was then used as a template for messenger RNA (mRNA) amplification on Bio‐Rad CFX96 real‐time PCR (Bio‐rad Company), including TNF‐α, IL‐1β, IL‐6, and IL‐17. RT‐PCR reaction conditions: predenaturation at 95°C for 30 s, 39 cycles of denaturation at 95°C for 5 s, annealing at 60°C for 5 s, and extension at 65°C for 5 s. The primers were listed in Supporting Information: Table 1. Glyceraldehyde‐3‐phosphate dehydrogenase was used as an internal reference and relative expression levels were calculated by using the $2^{‐\Delta \Delta C_{T}}$ method.

### 2.11 Western blotting

Mouse skin tissues in each group were collected and lysed into tissue homogenates by adding RIPA lysis solution containing protease inhibitors to extract total proteins. Protein concentrations were determined using the BCA protein assay kit (Thermofisher) and subsequently electrophoresed using 8%–12% sodium dodecyl sulfate‐polyacrylamide gels. A semidry method was employed to transfer proteins to nitrocellulose membranes, and then blocking was subsequently performed using 1% bovine serum albumin. Primary antibodies were diluted by phosphate buffered saline (PBS) solution with the detergent Tween® 20 (PBST) to working solution concentrations and incubated with proteins overnight. Secondary antibodies were diluted by PBST to working solution concentration after incubating proteins for 1 h in a constant temperature shaker protected from light at 37°C. The gray values of protein bands were finally scanned with ImageJ setup software. The information of antibodies was listed in Supporting Information: Table 2. The relative level of protein expression was expressed as the ratio of the gray value of the protein band of interest to the gray value of the internal reference protein.

### 2.12 Statistical analysis

Data are processed using SPSS 22.0 statistical software and presented as the mean ± standard error of mean (SEM) of results from at least three independent experiments. Differences among more than two groups in the above assays were estimated using one‐way analysis of variance, with *p* < .05 considered significant.

## 3 RESULTS

### 3.1 THL ameliorated IMQ‐induced psoriasis‐like skin lesions in mice

**Figure 1:**
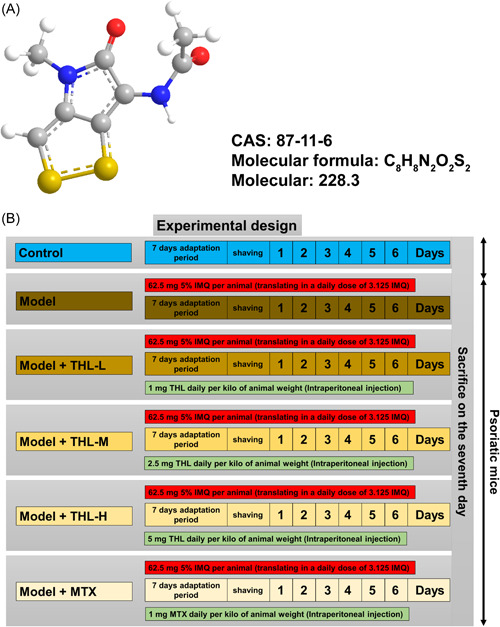
Chemical structure of thiolutin (THL) and the experimental design. (A) The chemical structure formula, molecular formula, CAS number, and molecular mass of THL. (B) Illustration of experimental design. D −7 to D −1: mouse adaptation period. D +0: shaving. D +1 to D +7: imiquimod (IMQ) modeling + different treatments. D +7: killed, samples were collected for further biochemical measurements.

**Figure 2:**
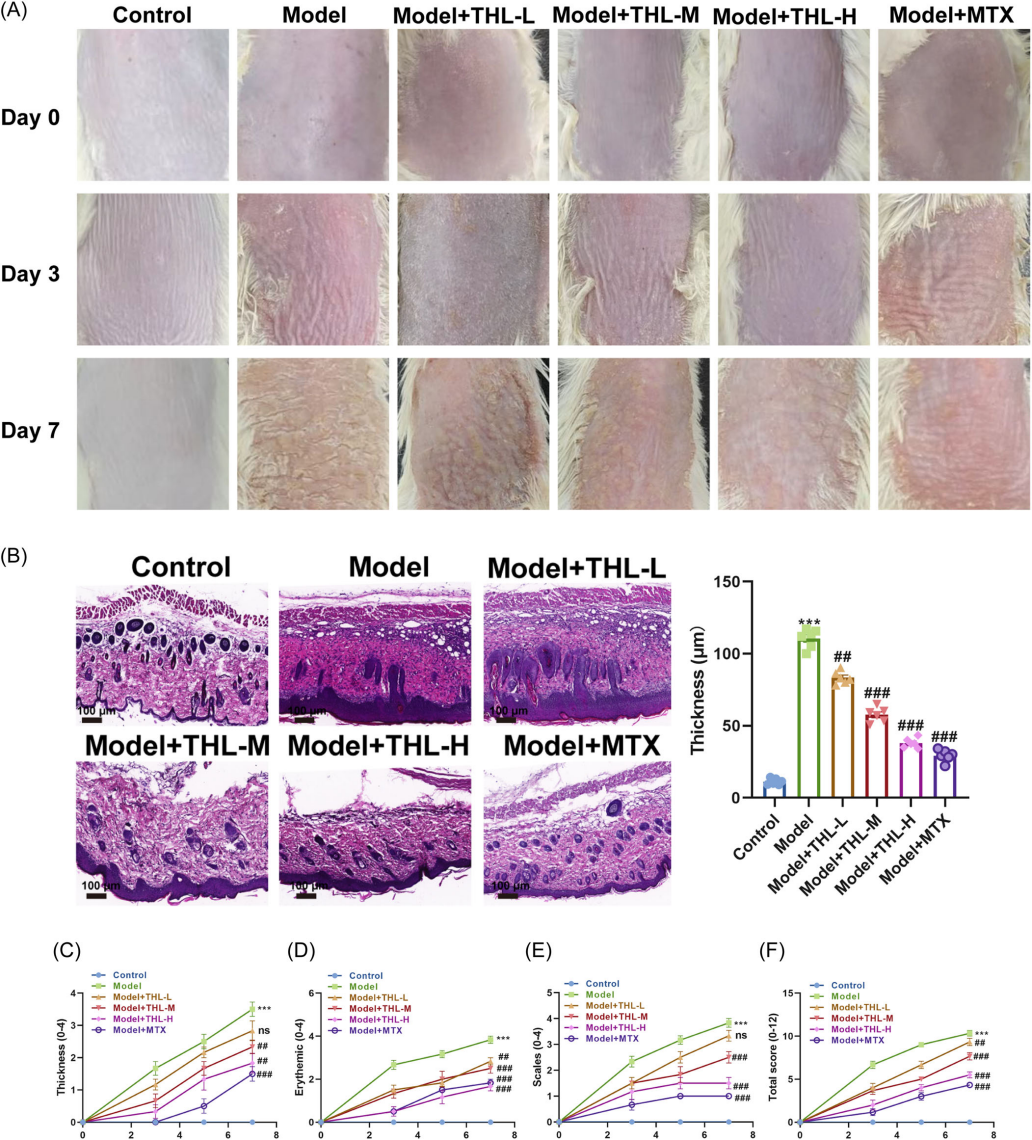
Thiolutin (THL) ameliorated imiquimod‐induced psoriasis‐like skin lesions in mice. (A) Representative pictures of the back of mice at 0, 3, and 7 days after THL intervention. (B) Left‐hematoxylin and eosin staining observation of the pathological changes in the lesional skin of mice on the 7th day after THL intervention (scale bar = 100 μm; original magnification = ×100); Right‐epidermal thickness measurement. (C–F) Psoriasis area and severity index. Scoring criteria: 0—none; 1—slight; 2—moderate; 3—marked; 4—very marked. The data represent mean ± SEM (*n* = 6 mice per group), *p* values were obtained by one‐way analysis of variance. ***p ＜ .001 versus control group; ^#^ *p* ＜ .05; ^##^ *p* ＜ .01; ^###^ *p* ＜ .001 versus model group.

Figure 1A is shown as the chemical structural formula of THL, and the chemical abstracts service (CAS) number of THL is 87‐11‐6. The mice were randomly divided into six groups (*n* = 6) as follows: control, model, model + THL‐L, model + THL‐M, model + THL‐H, and model + MTX groups (Figure 1B). Mice were photographed and scored at 0, 3, and 7 days after modeling. As shown in Figure 2A, the mice in the control group all had delicate and smooth skin. IMQ‐induced mice developed scaling involving the entire skin surface with subtle redness of the skin over 3 days of modeling. At 7 days, the scale was more densely distributed, the thickening phenomenon was further aggravated, and the erythema color was further deepened. The phenomena of erythema, scaling, and thickening of the mouse skin were materially improved after intervention in different dosage groups of THL and MTX positive control group. Among them, the THL high‐dose group showed the most significant effect, with a more obvious improvement compared with the MTX positive control group. Subsequently, the mice skin tissues were subjected to HE staining, and the skin histopathological changes were observed under a light microscope. As shown in Figure 2B, the skin tissue structure of the control group mice was normal, with only two or three layers of epidermal layer cells. Compared with the mice in the control group, IMQ‐induced mice displayed obvious thickening of the epidermis, with capillary hyperplasia and inflammatory cell infiltration in the superficial layer, parakeratosis in the stratum corneum, thinning or even disappearance of the stratum granulosum, and acanthosis with epidermal protrusion extension. Compared with the model group, the epidermal thickness of mice in the THL (1, 2.5, and 5 mg/kg) and MTX (1 mg/kg) groups was conspicuously thinner, and the parakeratosis, capillary proliferation, and inflammatory cell infiltration were significantly reduced, with the greatest ameliorative effect observed in the THL‐H group. Next, the erythema, scales, and thicknesses of the shaved sites of mice at D0, D3, D5, and D7 were scored according to the PASI scoring criteria, and the total score was calculated, which was plotted. The results demonstrated that the erythema, scales, thicknesses, and total score of mice in the control group were 0. The skin scale, thickening, erythema, and total score of IMQ‐induced model mice continuously increased at D0–D7, and the total score reached 11 at D7. The skin scale, thickening, erythema, and total score of mice after THL (1, 2.5, 5 mg/kg) and MTX (1 mg/kg) interventions also continuously increased at D0–D7, but the values were lower than those of the model group, in which the THL at 5 mg/kg was close to that of the positive control MTX group (Figure 2C).

### 3.2 THL did not affect liver and kidney function

**Figure 3:**
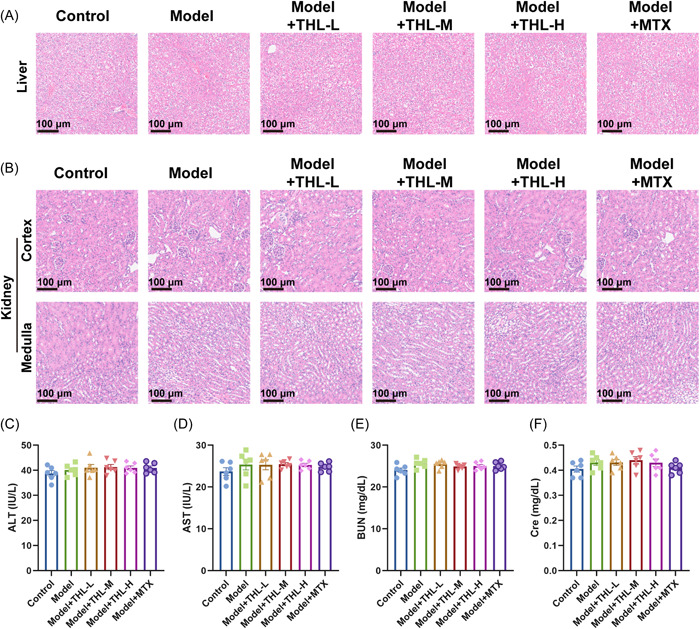
Thiolutin (THL) did not affect liver and kidney function. (A) Hematoxylin and eosin (HE) staining observation of the pathological changes in the liver of mice on the 7th day after THL intervention (scale bar = 100 μm; original magnification = ×200). (B) HE staining observation of the pathological changes in the cortex and medulla of mice kidney on the 7th day after THL intervention (scale bar = 100 μm; original magnification = ×200). (C) Small animal fully automatic biochemical analyzer detection of alanine aminotransferase (ALT) content in serum after THL intervention. (D) Small animal fully automatic biochemical analyzer detection of aspartate aminotransferase (AST) content in serum after THL intervention. (E) Small animal fully automatic biochemical analyzer detection of blood urea nitrogen (BUN) content in serum after THL intervention. (F) Small animal fully automatic biochemical analyzer detection of blood creatinine (CREA) content in serum after THL intervention. The data represent mean ± SEM (*n* = 6 mice per group), *p* values were obtained by one‐way analysis of variance.

Psoriasis is a common recurrent skin disease, many drugs used clinically to manage psoriasis can damage liver and kidney functions, such as MTX. Therefore, we next further evaluated the liver and kidney functions in mice after THL intervention to explore whether THL would cause damage to the liver and kidney. The results of HE staining of liver and kidney sections displayed that there were no significant changes in the histopathology of the liver (Figure 3A) and kidney (Figure 3B) in mice in each group after THL intervention. Subsequently, we further examined liver function evaluation indexes, including ALT (Figure 3C) and AST (Figure 3D), as well as renal function evaluation indexes, including BUN (Figure 3E) and CREA (Figure 3F) in mice. The results presented in Figure 3C–F, the indexes of ALT, AST, BUN, and CREA in each group showed no significant changes, which suggested that the treatment of psoriasis by THL intervention did not affect the liver and kidney functions.

### 3.3 THL attenuated IMQ‐induced skin inflammation

**Figure 4:**
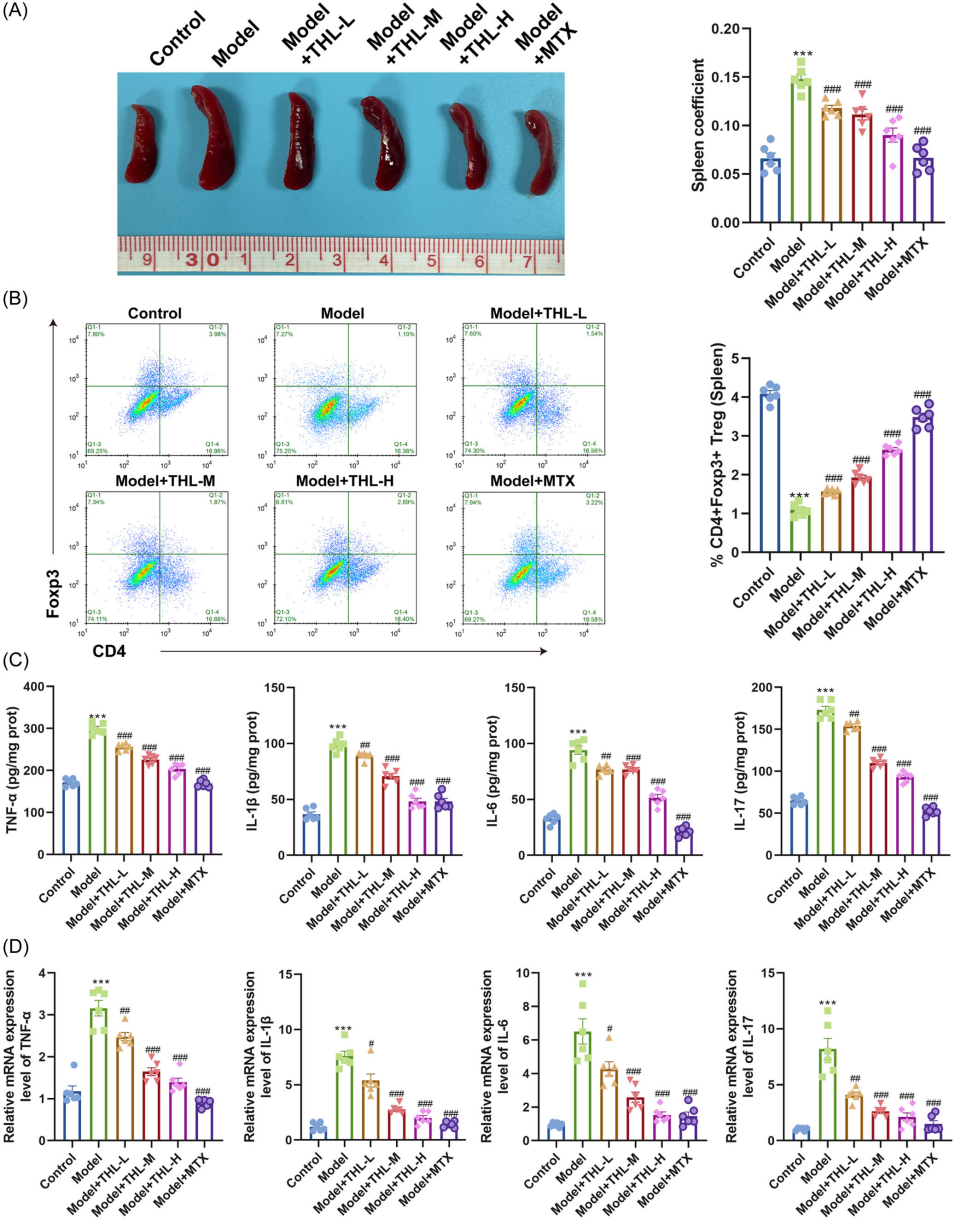
Thiolutin (THL) attenuated imiquimod‐induced skin inflammation. (A) Effect of THL on spleen changes and spleen index in psoriatic mice. (B) Flow cytometry detection of the proportion of Foxp3 + CD4 + cells in spleens of mice after THL intervention. (C) Enzyme‐linked immunosorbent assay detection of the contents of tumor necrosis factor‐α (TNF‐α), interleukin (IL)‐1β, IL‐6, and IL‐17 in mouse serum after THL intervention. (D) Reverse transcription‐quantitative polymerase chain reaction detection of the messenger RNA (mRNA) levels of TNF‐α, IL‐1β, IL‐6, and IL‐17 in mouse skin tissues after THL intervention. The data represent mean ± SEM (*n* = 6 mice per group), *p* values were obtained by one‐way analysis of variance. **p ＜ .01; ***p ＜ .001 versus control group; ^#^ *p* ＜ .05; ^##^ *p* ＜ .01; ^###^ *p* ＜ .001 versus model group.

The spleen is the largest immune organ of the human body and contains a large number of immune cells, while the body produces inflammatory responses to a certain extent and the immune system has a great connection. We removed the spleen of mice in each group, and the spleen index was calculated according to the formula. The results revealed that compared with the mice in the control group, the spleens of the mice in the model group were strikingly enlarged, and the spleen index was signally enlarged. Moreover, the phenomenon of splenomegaly was alleviated and the spleen index was observably reduced in the THL‐L/M/H treated mice, with the most significant effect in the THL‐H group, which was close to that in the positive control MTX group (Figure 4A). It has been well established that CD4 + Foxp3 Treg cells play an indispensable role in suppressing autoimmune inflammatory responses, including psoriasis.^21^ The flow cytometry results suggested that the number of CD4 + Foxp3 Treg cells in the spleen of mice was significantly decreased after IMQ‐induction, whereas the CD4 + Foxp3 Treg cell number was increased in a dose‐dependent manner after different doses of THL intervention and gradually approached that of the MTX‐treated group (Figure 4B). It is well established that cytokines TNF‐α, IL‐1β, IL‐6, and IL‐17 are involved in tissue inflammation, especially in skin inflammation associated with psoriasis, and can interact with immune cells to exert proinflammatory effects. In accordance with this, we further evaluated proinflammatory factor levels in mouse serum and skin tissues by RT‐qPCR and ELISA. Figure 4C results showed that the contents of TNF‐α, IL‐1β, IL‐6, and IL‐17 in the serum of mice after IMQ‐induced modeling were significantly increased, while THL intervention dose‐dependently decreased the production of above proinflammatory factors, among which the THL‐H group was the closest to the MTX therapy group (Figure 4C). Similarly, RT‐qPCR results displayed that the mRNA levels of TNF‐α, IL‐1β, IL‐6, and IL‐17 were conspicuously upregulated in the model group, which were gradually decreased by THL intervention in a dose‐dependent manner (Figure 4D).

### 3.4 THL inhibits NLRP3 inflammasome activation in IMQ‐induced skin

**Figure 5:**
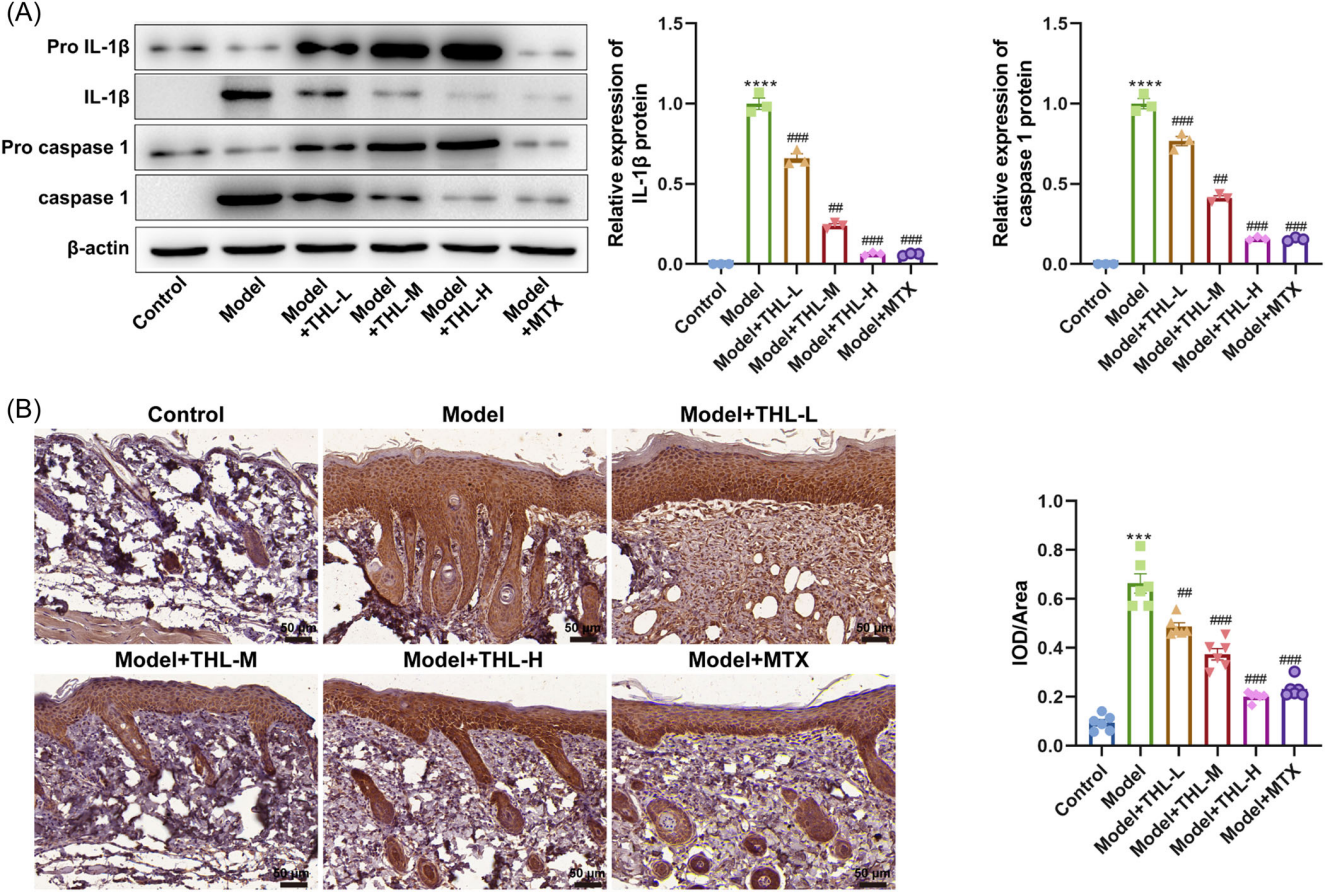
Thiolutin (THL) inhibited NOD‐like receptor protein 3 (NLRP3) inflammasome activation in imiquimod‐induced skin. (A) Western blotting detection of the protein levels of interleukin‐1β and caspase‐1 precursor and maturases in the skin tissues of mice after THL intervention. The data represent mean ± SEM (*n* = 3), *p* values were obtained by one‐way analysis of variance (ANOVA). (B) Immunohistochemistry detection of the expression and localization of NLRP3 in the skin tissues of mice after THL intervention (scale bar = 50 μm; original magnification = ×100). The data represent mean ± SEM (*n* = 6 mice per group), *p* values were obtained by one‐way ANOVA. ****p ＜ .0001 versus control group; ^##^ *p* ＜ .01; ^###^ *p* ＜ .001 versus model group.

Next, we further explored whether the regulation of THL on IMQ‐induced psoriasis‐like symptoms in mice was associated with NLRP3 inflammasome activation. Western blotting was applied to examine the protein expression of IL‐1β and caspase‐1 precursor and maturases in the skin tissues of mice in each group, and the results showed that the protein levels of IL‐1β and caspase‐1 maturases were largely absent in the control mice, whereas the levels of the IL‐1β and caspase‐1 precursor proteins were strikingly downregulated, whereas the expression of the mature precursor protein was prominently increased, in the skin tissues of the model mice. Moreover, IL‐1β and caspase 1 precursor protein levels were dose‐dependently upregulated after different doses of THL intervention, whereas mature precursor protein expression was gradually decreased (Figure 5A). In addition, we detected the expression and localization of NLRP3 in skin tissues by using immunohistochemistry. The results suggested that NLRP3 was expressed in some parts of the epidermal cells in the control group with weak staining, whereas NLRP3 was strongly expressed in the cytoplasm of the full‐thickness cells of the epidermis in the IMQ‐induced mouse psoriasis‐like model group. Furthermore, the mice that received an intraperitoneal injection with different doses of THL showed a gradual attenuation of NLRP3 expression compared with the model group, in which the NLRP3 staining intensity was the weakest between the THL‐H group and the MTX group, suggesting that 5 mg/kg of THL can significantly inhibit NLRP3 expression and the effect is close to that of MTX treatment (Figure 5B). The above results confirmed that THL was able to target NLRP3 inflammasome to repress its expression and activation.

## 4 DISCUSSION

Psoriasis is a chronic inflammatory skin disease and recurrent, which is characterized by immune cell infiltration and inflammatory responses.^22^ Many studies found that topical application of IMQ to the exposed dorsal skin of mice for 5–7 days resulted in psoriasiform lesions.^23^, ^24^ The IMQ‐induced mouse model has very similar pathological features to human psoriasis, the mouse epidermis presents diffuse hyperplasia and inflammatory cell infiltration, which is economical and convenient compared with other models, and the anti‐inflammatory effects of most natural drugs have been demonstrated in IMQ‐induced psoriasis.^25^ Therefore, IMQ was chosen to induce the establishment of a psoriasis model in mice in this study. Psoriasis, as a recurrent skin disease, can be divided into the following clinical treatment methods: oral medicine, external medicine, and physical therapy. The side effects of these drugs also vary, and oral and topical drugs are often accompanied by swelling, stinging, and pruritus.^26^ Physical therapy has greatly increased the incidence of skin cancer.^27^ Emerging biologics are expensive and therefore their clinical use is limited.^28^ Therefore, it is of great significance to seek for economical and less‐side effects drugs for clinical treatment of psoriasis. THL is a disulfide containing antibiotic and antiangiogenic compound whose antitumor activity is currently demonstrated. Minamiguchi et al.^29^ revealed that THL could effectively inhibit the adhesion of HUVECs to vitronectin, which in turn conspicuously inhibited angiogenesis induced by tumor cells. Jing et al.^30^ found that THL as an inhibitor of PSMD14 was able to prevent neck squamous cell carcinoma tumor growth. Nevertheless, the role of THL in inflammatory‐related diseases has rarely been studied. This study first evaluated the therapeutic effects of THL on the IMQ‐induced psoriasis model in mice at the overall level. The results showed that THL significantly reduced the thickness, scaling, and erythema of the skin lesions, alleviated IMQ‐induced psoriasiform lesions in mice, reduced the PASI score and ameliorated IMQ‐induced histopathological changes in mouse skin, demonstrating that THL could reduce symptoms of psoriasiform dermatitis and has therapeutic effects on psoriasis. Besides, a recent study found that THL treatment attenuated the inflammatory invasion of several inflammatory diseases, such as nonalcoholic fatty liver induced by methionine choline‐deficient diet.^20^ These results initially revealed that THL has a better therapeutic effect on the IMQ‐induced psoriasis model.

In addition to the characteristic psoriatic lesions in the skin, the IMQ‐induced psoriatic mouse model exhibits several systemic inflammatory responses, including enlargement of the spleen and lymph nodes as well as changes in immune T and B lymphocytes in the lymph nodes.^31^ The spleen is an important immune organ in the human body and can produce a variety of cells, including lymphocytes, monocytes, and plasma cells, which play a crucial role in innate and adaptive immune responses.^32^ In this study, the spleen index of mice in the model group was elevated more than twofold compared with that in the control group, indicating that IMQ can hyperactivize the immune system. The spleen index of mice was decreased by nearly half after intervention via THL‐H group, which was similar to the results of MTX group, revealing that THL could inhibit immune activity. Furthermore, the main immunoregulatory cells CD4 + T lymphocytes include helper T lymphocytes (Th)1 cells, Th2 cells, Th17 cells, and Treg cells, which are capable of producing different biological functions and roles, respectively, among which Treg cells are “new stars” among CD4 + T lymphocytes.^33^ Foxp3 has demonstrated the ability to regulate the differentiation and transcription of Treg cells, reflecting the activation ability of Treg cells.^34^ While the expression of Th17/Treg cells in normal individuals is in equilibrium, in patients with psoriasis the two are out of balance and the expression activity of Treg cells is diminished.^35^ A previous study found that Foxp3 in skin tissue is mainly expressed on CD4 + T cells and is therefore considered a characteristic marker for in situ recognition of Treg cells. Therefore, the study of the proportion of Foxp3 + CD4 + Treg cells represents an entry point for the investigation of the pathogenesis of psoriasis and the treatment with targeted agents. Zhao et al.^36^ suggested that the proportion of Foxp3 + CD4 + Treg cells was significantly reduced in patients with psoriasis, and increasing Foxp3 expression was able to improve immune dysfunction in CD4 + T cells from patients with psoriasis. Our results displayed that the number of CD4 + Foxp3 Treg cells in the spleen of mice was materially decreased after IMQ‐induction, whereas the number of CD4 + Foxp3 Treg cells was increased in a dose‐dependent manner after different doses of THL intervention and gradually approached that of MTX treatment. After that, we detected the expression of proinflammatory factors TNF‐α, IL‐1β, IL‐6, and IL‐17 in the skin of IMQ‐induced psoriatic mice by RT‐qPCR and ELISA, and found that THL could decrease the expression levels of TNF‐α, IL‐1β, IL‐6, and IL‐17 in the skin of psoriatic mice, indicating that it ameliorates skin inflammation. Additionally, at present, there is an increasing number of studies on the hepato‐ and nephrotoxic damage caused by MTX, and many clinical claims have been made to reduce the dosage of MTX, thereby reducing its damage to liver and kidney function.^37^ Cabello Zurita et al.^38^ performed a retrospective review and found that liver (2.8%) or kidney (0.5%) abnormalities were seen in 3.3% of psoriatic patients treated with MTX. A previous study showed that THL was able to effectively attenuate nonalcoholic fatty liver‐mediated liver function injury, suggesting a possible hepatoprotective effect.^20^ In the present study, it was found that THL intervention for psoriasis did not affect liver and kidney function, which reflects its safety.

NLRP3 belongs to the nod‐like receptors family, an intracellular PRR of the innate immune system, which are expressed on a variety of cells such as macrophages, dendritic cells, neutrophils, keratinocytes, and epithelial cells.^39^ After NLRP3 recognizes pathogen‐associated molecular patterns or danger‐associated molecular patterns, it forms a functional complex with the adaptor protein ASC and the cysteine protease caspase‐1 in the cytosol, the NLRP3 inflammasome.^40^ A previous study revealed that IMQ‐activated the production of adenosine triphosphate (ATP), inducible nitric oxide synthase (iNOS), reactive oxygen species (ROS), and other factors in an induced mouse psoriasis‐like model.^41^, ^42^ Whereas ATP and ROS can encourage NLRP3 to oligomerize and recruit ASC and caspase‐1 precursor, which in turn activates to form caspase‐1 mature. Caspase‐1 is subsequently cleaved to the IL‐1β precursor, which in turn generates the active IL‐1β mature.^43^ Su et al.^44^ demonstrated that the expression of NLRP3 in psoriatic samples was 3.5‐ to 4.3‐fold higher than that in normal skin biopsies, with concomitant elevated IL‐1β and caspase‐1 expression. Similarly, we found that the expression levels of NLRP3, caspase‐1, and IL‐1β in the psoriatic‐like lesions of model mice treated with topical IMQ were all increased compared with normal control mice, which was consistent with previous findings. In addition, THL intervention particularly reduced the expression levels of NLRP3, caspase‐1, and IL‐1β, suggesting that THL has an active inhibitory effect on NLRP3 inflammasome. Chen et al.^45^ found that chlorquinaldol, a historic antimicrobial agent, could inhibit the activation of NLRP3 as a target and then reduce the severity of psoriasis reaction in vivo. From this, we speculated that THL might target the NLRP3 inflammasome as a drug target. Interestingly, THL has been demonstrated to inhibit BRC3 containing isopeptidase complex‐mediated NLRP3 de‐ubiquitination and activation in a previous study by Ren et al.^20^ This may be the mechanism and basis for THL to target NLRP3 as a drug.

The above studies speculated that NLRP3 inflammasome has a role in the pathogenesis of IMQ‐induced psoriasis‐like lesions in mice, and THL may alleviate psoriasis‐like manifestations in mice by inhibiting NLRP3 inflammasome.

However, some limitations exist in this study. First, the route of administration of psoriasis in a clinic is mostly topical application, so we will further explore the therapeutic effect based on psoriasiform skin inflammation through the topical route of administration in subsequent experiments. Besides, whether the mechanism of THL involved in the regulation of psoriasis is mainly mediating the deubiquitination of NLRP3 as previously discussed awaits further validation by subsequent experiments. In summary, the study herein provides a new drug candidate for the treatment of psoriasiform skin inflammation, and sheds new light on the subsequent research of psoriasis.

## AUTHOR CONTRIBUTIONS

**Aixue Wang**: Conceptualization; data curation; formal analysis; methodology; investigation. **Xixing Ma**: Data curation; formal analysis; methodology. **Feng Wei**: Data curation; formal analysis. **Yanling Li**: Data curation; formal analysis. **Qiang Liu**: Data curation; formal analysis. **Huanhuan Zhang**: Conceptualization; formal analysis; methodology; writing—original draft; writing—review and editing. All authors have read and approved the final manuscript.

## CONFLICT OF INTEREST STATEMENT

The authors declare no conflict of interest.

## ETHICS STATEMENT

All animal experimental protocols were reviewed and approved by the institutional animal care committee of the Second Affiliated Hospital of Hebei Medical University.

## Supporting information

Supporting information.

## Data Availability

All data generated or used during the study appear in the submitted article.
