# Supplementary material for: Evidence on the therapeutic role of thiolutin in imiquimod‐induced psoriasis‐like skin inflammation in mice
Source: Immun Inflamm Dis. 2023 Jul 12;11(7):e877. doi: 10.1002/iid3.877 (PMC10336655; doi:10.1002/iid3.877)
Supplement: Supplementary file 1 — Supporting information. [file IID3-11-e877-s001.docx]

**Supplementary Table 1. Primer sequences**

| **Gene** | **Sequence** |  |
| --- | --- | --- |
| TNF-α | Forward | CTCCACTTGGTGGTTTGCTAC |
|  | Reverse | CTTCCCTCTCATCAGTTCTATGG |
| IL-1β | Forward | GAAGAAGAGCCCATCCTCTGT |
|  | Reverse | TGTTCACGGAGCCTGTAG |
| IL-6 | Forward | ACCACTCCCAACAGACCTGTCT |
|  | Reverse | CAGATTGTTTTCTGCAAGTGCAT |
| IL-17 | Forward | CTGAACATCCATAACCGGAATACCA |
|  | Reverse | AGCGTTGATGCAGCCCAAG |
| GAPDH | Forward | ACAACTTTGGTATCGTGGAAGG |
|  | Reverse | GCCATCACGCCACAGTTTC |

**Supplementary Table 2. The antibodies used in this research**

| **Antibody** | **Manufacturer** | **Cat.no** |
| --- | --- | --- |
| Pro IL-1β | Abcam | ab216995 |
| IL-1β | Bioss Inc. | bs-6319R |
| Pro caspase1 | Abcam | ab179515 |
| caspase1 | NSJ Bioreagents | R30511 |
| β-actin | Abcam | ab8226 |
